# Organotypic Culture of Breast Tumor Explants as a Multicellular System for the Screening of Natural Compounds with Antineoplastic Potential

**DOI:** 10.1155/2015/618021

**Published:** 2015-05-17

**Authors:** Irma Edith Carranza-Torres, Nancy Elena Guzmán-Delgado, Consuelo Coronado-Martínez, José Inocente Bañuelos-García, Ezequiel Viveros-Valdez, Javier Morán-Martínez, Pilar Carranza-Rosales

**Affiliations:** ^1^Centro de Investigación Biomédica del Noreste, Instituto Mexicano del Seguro Social, 64720 Monterrey, NL, Mexico; ^2^Facultad de Ciencias Biológicas, Universidad Autónoma de Nuevo León, 64460 San Nicolás de los Garza, NL, Mexico; ^3^Unidad Médica de Alta Especialidad No. 34, Instituto Mexicano del Seguro Social, 64730 Monterrey, NL, Mexico; ^4^Unidad Médica de Alta Especialidad No. 23, Instituto Mexicano del Seguro Social, 64010 Monterrey, NL, Mexico; ^5^Facultad de Medicina, Universidad Autónoma de Coahuila, 66451 Torreón, COAH, Mexico

## Abstract

Breast cancer is the leading cause of death in women worldwide. The search for novel compounds with antitumor activity, with less adverse effects and higher efficacy, and the development of methods to evaluate their toxicity is an area of intense research. In this study we implemented the preparation and culture of breast tumor explants, which were obtained from precision-cut breast tumor slices. In order to validate the model we are proposing to screen antineoplastic effect of natural compounds, we selected caffeic acid, ursolic acid, and rosmarinic acid. Using the Krumdieck tissue slicer, precision-cut tissue slices were prepared from breast cancer samples; from these slices, 4 mm explants were obtained and incubated with the selected compounds. Viability was assessed by Alamar Blue assay, LDH release, and histopathological criteria. Results showed that the viability of the explants cultured in the presence of paclitaxel (positive control) decreased significantly (*P* < 0.05); however, tumor samples responded differently to each compound. When the explants were coincubated with paclitaxel and compounds, a synergic effect was observed. This study shows that* ex vivo* culture of breast cancer explants offers a suitable alternative model for evaluating natural or synthetic compounds with antitumor properties within the complex microenvironment of the tumor.

## 1. Introduction

Cancer is the leading cause of mortality worldwide, with 8.2 million deaths and 14.1 million new cases recorded during 2012 alone. According to the World Health Organization, the number of deaths will continue to rise across the globe, with the alarming prediction of 19.3 million new cases by 2025. Breast cancer is the most frequent cancer found in women; it possesses the most elevated morbidity and mortality. In 2012, approximately 1.7 million women were diagnosed with breast cancer in the world, and 522,000 died as a direct result of this disease [1].

Conventional cancer therapies include surgery, radiation, and chemotherapy. Although the latter is widely used, in most cases it produces undesirable side effects. Chemoresistance and/or recurrence of cancer after chemotherapy are frequent events seen with treatment of this disease [2]. Thus, different research groups are now focused on finding novel drugs or anticancer compounds [3, 4] while others are developing methodologies for the evaluation of these drugs [5–7].

One of the current approaches for investigating novel antineoplastic or chemopreventive compounds is based on natural products research. This is because some of these compounds inhibit cell proliferation and promote apoptosis in various types of tumor cells including breast cancer cells. Furthermore, it is well known that approximately 60% of the drugs administered in cancer treatment were isolated from natural products [8–10].

For the purpose of this study, we selected three naturally occurring compounds which possess antitumor and chemopreventive activities, namely, caffeic acid (CA), ursolic acid (UA), and rosmarinic acid (RA). These bioactive compounds are present in fruits, vegetables, medicinal plants, and culinary species.  Table 1 shows their chemical structures and some examples of culinary herbs and spices where they are abundantly found [11–13]. CA is known to inhibit DNA methylation in human breast cancer cells and it has been suggested that it may reduce the risk of acquiring breast cancer [14]; however, epidemiological studies have been inconsistent and no established association between coffee intake and breast cancer development has been discovered [15]. We have previously reported that CA obtained from* Hedeoma drummondii* extracts possesses antiproliferative effect against MCF-7 and HeLa cells [16]. UA is known to inhibit proliferation of MCF-7 cells [17, 18], induce apoptosis, and inhibit oxygen consumption in various tumor cell lines [19, 20]. It suppresses the migration and invasion of MDA-MB-231 cells [21] and exerts antitumor effects on multidrug-resistant cancer cells [22]. RA has antioxidant, antitumor, antimutagenic, and chemopreventive activities [12, 23–25]. The effect of RA on cell proliferation and apoptosis induction has been determined in MCF-7, MDA-MB-361, MDA-MB-453, and HeLa cells [26, 27]; RA induces apoptosis and inhibits metastasis of MDA-MB-231BO cells. Hence, RA is also considered a good candidate for new therapeutic approaches in the treatment of breast cancer [28].

On the other hand, the antitumor activity and mechanisms involved in the inhibition of carcinogenesis by novel compounds with antineoplastic potential must be evaluated and validated using models that extrapolate their effects in humans. The results obtained from cells cultured* in vitro *and from experiments conducted on animals do not reflect what happens in humans, especially with regard to the full physiology, metabolism, pharmacokinetics, and other factors of high complexity. Therefore, it is important to use experimental models for easy and proper observation of the effects of bioactive compounds in tumor samples where tumor microenvironment is preserved. Conde et al. suggest that to study behavior of tumors it is necessary to maintain or reconstitute a similar environment of the tumor* in situ* [29]. From the experimental point of view, a way to preserve tissue architecture with little or no manipulation is through the organotypic culture of intact and fresh tumor tissues. Tissue slices, one of the methods recently used, is an intermediate system between* in vivo* and* in vitro* models, which offers a new perspective to the results obtained with cell lines.

Tissue slices contain virtually all the cells from the tissue under study. They retain histological and three-dimensional structure (3D), with inter- and extracellular interactions, cell matrix components, and, most interestingly, metabolic capacity. Hence, cultured tissue slices are considered a suitable tool for the study of multicellular processes [30].

Precision-cut tissue slices have mainly been used to study metabolism and toxicity of xenobiotics [31, 32], biotransformation of drugs, gene expression studies, and morphological analysis, among other studies [33, 34]. Our group has recently described their application as an infection model for the parasitic protozoa* Entamoeba histolytica* [35, 36]. Other than normal tissue slices, tumor slices are 3D cultures in which it is possible to evaluate* ex vivo* therapeutic efficacy of oncolytic vectors [37–41] and drugs such as meloxicam and Taxol [42–44] or study the interactions between stromal components and epithelial cells with the extracellular environment, as well as the response to cytokines and drugs [45]. The taxoid paclitaxel (abbreviated TX in this work), known by its original brand name, Taxol, represents the most important first-line antineoplastic drug for treatment of various types of cancer, including breast cancer, ovarian cancer, non-small cell lung cancer, and AIDS related Kaposi's sarcoma, among others. TX was purified and identified as the active constituent from the bark of the Pacific yew,* Taxus brevifolia*, in 1971 [46, 47]. Its mechanism of action relies on the promotion of microtubule assembly and inhibition of microtubule disassembly; cells exposed to paclitaxel cannot form a mitotic spindle; this interferes with cell division and induces cell death [48].

Using* ex vivo* organotypic cultures of breast cancer explants treated with CA, UA, RA, and TX, we found that this model is an alternative system for studying anticancer activity or synergistic potential assessing natural products. Cultured explants retain their typical morphology and viability for at least 3 days. With this method, a sufficient number of slices and explants can be obtained from minimal amounts of tissue, enabling the study of several compounds within a single tumor specimen.

## 2. Materials and Methods 

### 2.1. Chemicals

Caffeic acid, ursolic acid, rosmarinic acid, paclitaxel, and insulin-transferrin-selenium were purchased from Sigma-Aldrich (St. Louis, MO, USA). DMEM/F12 medium, fetal bovine serum, gentamicin, penicillin-streptomycin, and Alamar Blue were obtained from Invitrogen (Grand Island, NY, USA). The antibody against Ki 67 was obtained from Santa Cruz Biotechnology (Santa Cruz, CA, USA). The reagents for general use were purchased from Sigma-Aldrich (St. Louis, MO, USA).

### 2.2. Tumor Samples

Infiltrating ductal adenocarcinoma specimens were collected from 11 patients during surgery at the Hospital of Gynecology and Obstetrics (UMAE # 23) from the Mexican Institute of Social Security (IMSS). The pathologist dissected the specimen immediately after surgery to confirm its tumorous nature and to avoid contamination. Informed consent was obtained from all patients. Tissues were collected in cold serum-free DMEM/F12 medium (Invitrogen, Grand Island, NY, USA) and transported at 4°C to the organotypic culture laboratory for immediate processing. Approval was obtained from the Institutional Review Board (Mexican Institute of Social Security) before initiation of studies on human tissue. The clinical and histopathological data of these patients are described in Table 2.

### 2.3. Preparation of Slices and Explants from Breast Tumor

From representative tumor samples, cylindrical tissue cores of 10 mm diameter were obtained; from these, tissue slices of 250–300 *μ*m thickness were prepared using the Krumdieck tissue slicer (Alabama Research & Development, Munford, AL, USA), with constant flow of Krebs Henseleit bicarbonate buffer (KB) at 4°C which was gassed with carbogen. The slices were collected in KB buffer at 4°C. To optimize the tumor sample and homogenize the size of tissues, small tumor explants, 4 mm in diameter and 250–300 *μ*m in thickness, were prepared using a biopsy punch from the first-obtained slices. Tumor explants were placed in six-well microplates containing DMEM/F12 culture medium supplemented with 10% fetal bovine serum, 5 *μ*g/mL bovine insulin, 100 *μ*g/mL gentamicin, insulin-transferrin-selenium, and 25 mM glucose (DMEM/F12 supplemented medium). Plates were preincubated for 1 h at 37°C, 5% CO_2_/95% air, and agitation at 25 rpm. The interval between resection of the tumor and the incubation of the explants was no more than 2 h. The entire process was performed under aseptic conditions.

### 2.4. Viability of Tumor Explants

In order to confirm that the tumor samples were still viable during the entire experiments, the viability of the tumor explants was determined at different times before testing the antineoplastic effect of CA, UA, and RA. To test this, explants with 4 mm diameter/250–300 *μ*m thickness were placed in 24-well microplates containing 1 mL of DMEM/F12 supplemented medium and incubated for 4 days at 37°C, 5% CO_2_/95% air, and constant agitation of 25 rpm. Viability was determined every 24 h in a group of four explants. Protocols for metabolic viability (AB), cytotoxicity (LDH release), cellular proliferation (Ki 67 expression), and morphological integrity (histopathological analysis) are described in the corresponding section. The culture medium was changed every 24 h through 96 h, and each time viability, proliferation, and morphology were assessed.

### 2.5. Treatment of Tumor Explants with TX and Bioactive Compounds

After 1 h of preincubation, the tumor explants were transferred to 24-well microplates containing 1 mL of DMEM/F12 supplemented medium. Afterwards, the following compounds were added: 20 *μ*g/mL TX (positive control), 11–33 *μ*g/mL CA, 20–60 *μ*g/mL for RA and UA, and combinations of these compounds with TX. These concentrations were selected on the basis of IC_50_ values reported in cell lines [16, 20, 49]. Control group (100% viability) consisted of untreated explants, which were incubated only with culture medium. Afterwards, the microplate with the explants and their corresponding treatments were incubated for 48 h at 37°C, 5% CO_2_/95% air, and constant agitation at 25 rpm.

### 2.6. Alamar Blue Viability Assay

The effect of treatment with CA, UA, and RA on the viability of the tumor explants was assessed by the Alamar Blue assay. Alamar Blue (AB) is a blue nonfluorescent dye reduced to a pink-colored, highly fluorescent resorufin by metabolically active cells. It is known that viable cells reduce the microenvironment to a pink color, while dead or inactive cells do not change the original blue color of resazurin, the active ingredient of AB. After 48 h of incubation with compounds, as well as with cell culture medium and TX (controls), the explants were incubated for additional 4 h with 10% Alamar Blue in 500 *μ*L DMEM/F12 supplemented medium at 37°C in the conditions described earlier. Afterwards, 100 *μ*L was collected from each sample and transferred to a 96-well microplate. Fluorescence values were read using a multinode microplate reader (Synergy BioTek HT) at 530 nm excitation/590 nm emission wavelengths. The percentage of viability relative to control was calculated using the free software AbD Serotec fluorometric calculator for AB assays (http://www.abdserotec.com/colorimetric-calculator-fluorometric-alamarblue.html).

### 2.7. Lactate Dehydrogenase Assessment

Another way to assess the viability of the explants treated with the compounds and that of the untreated controls was by assessment of the leakage of the enzyme lactate dehydrogenase (LDH) into the supernatant of the culture medium [50]. The assay is based on the release of the cytosolic enzyme LDH into the media by cells with damaged plasma membranes [51]. The cytotoxicity induced by CA, UA, and RA on the tumor explants can be quantitatively determined by measuring the activity of this enzyme. The total amount of released enzyme was determined using an Architect C400 clinical chemical analyzer (Abbott).

### 2.8. Histopathological Analysis

After each experimental time point, the explants were fixed in 10% neutral formalin and then embedded in paraffin using the conventional histological technique. Tissue sections of 4 *μ*m were prepared on a microtome and mounted on glass slides. Afterwards, the slides were deparaffinized and stained with hematoxylin and eosin (H&E). Then permanent sections were prepared with coverslips and synthetic resin. The stained preparations were observed by a pathologist using a Zeiss Axiostar Plus Brightfield microscope. Morphological parameters analyzed in treated and control explants included necrosis, viable/damaged tumor cells, and inflammation. Representative photographs of all treatments were obtained with a 5.0 MP Moticam camera.

### 2.9. Immunohistochemistry for Ki 67 Expression

Analysis of Ki 67 expression was performed on paraffin sections using the Dako LSAB System-HRP methodology to assess the rate of cell proliferation from treated and untreated tumor explants. The procedure was performed according to the recommendations of the manufacturer. The expression of this marker is nuclear, and the proliferation index was defined as follows: low: expression in ≤10% of cells; intermediate: expression in 10–20% of cells; and high: expression in ≥20% of cells [52].

### 2.10. Statistical Analysis

Statistical analysis was performed with SPSS version 22.0 software. Quantitative data were expressed as mean and standard deviation. Differences in continuous variables with normal distribution were analyzed with Student's* t*-test or the Mann-Whitney *U* test for nonnormal distributions.

## 3. Results 

### 3.1. Characteristics of Patient Samples Used for the Preparation and Culture of Breast Cancer Tissue Explants

Eleven samples of human breast cancer with histopathological diagnosis of infiltrating ductal adenocarcinoma with nonspecific pattern were collected fresh from the operating room. Three samples were used to standardize and optimize the preparation of precision-cut breast tumor slices, and, from these, explants of a defined size and thickness (4 mm in diameter and 250–300 *μ*m thick) were obtained for* ex vivo* culture under controlled conditions. Three more samples were used to standardize the concentrations to test each of the bioactive compounds and TX. Three other samples were included in three independent assays to assess the effect of CA, UA, RA, and TX on the viability of tumor tissue explants. The last two samples were discarded due to abundant necrosis, as well as elevated adipose and fibrous tissues, which prevented proper processing.

### 3.2. Viability of Tumor Explants

#### 3.2.1. Metabolic Activity: Alamar Blue Assay

**Figure 1:**
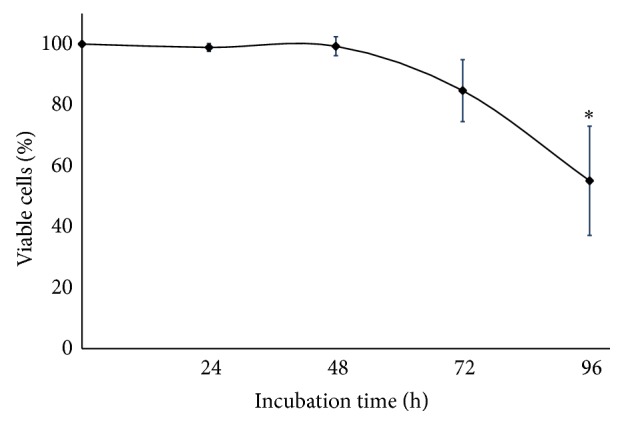
Viability of cultures breast cancer explants. To determine the optimal time for performing the experiments with bioactive compounds, explants were cultivated in DMEM/F12 supplemented medium for 24, 48, 72, and 96 h. The viability was assessed by the Alamar Blue assay. Values reflect means ± SD. Asterisks (∗) indicate significant statistical differences (*P* < 0.05).

Tumor explants cultured for 24 and 48 h remained viable throughout the incubation period, with mean viability of 99% compared to basal value (100%). At 72 h, a slight decrease was observed in viability (84.7% ± 10.2), whereas, at 96 h, the percentage decreased to 55.1% ± 17.9 (*P* < 0.05). These results showed the metabolism of explants of breast tumors, and hence, their viability remained intact during at least the first 48 h of culture. With these results, we decided that 48 h was the optimal time to perform cytotoxicity assays with the bioactive compounds (Figure 1).

#### 3.2.2. Morphological Integrity: Histopathological Analysis

**Figure 2:**
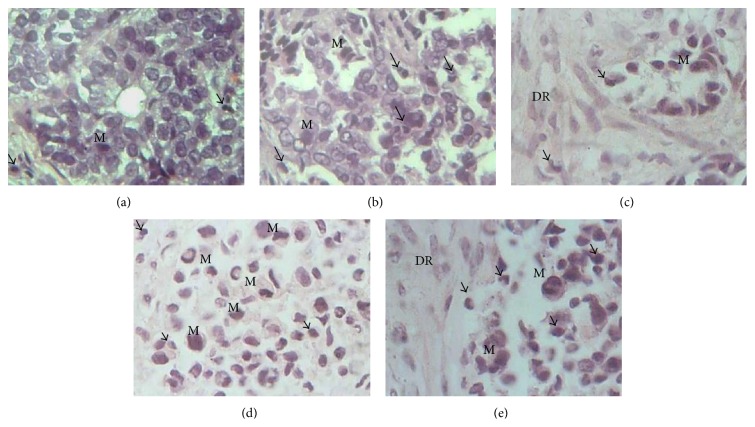
Morphological integrity of tumor tissue explants cultured* ex vivo*. Histopathological findings of tumor tissue at zero time (basal) and cultivated during different times in DMEM/F12 supplemented medium show that neoplastic tissue remains viable. It is possible to observe cells in active mitosis (M), desmoplastic reaction (DR) adjacent to the tumor cells, and presence of inflammatory cells (arrow). Desmoplasia or desmoplastic reaction is usually only associated with malignant neoplasms, which can evoke a fibrosis response by invading healthy tissue. All these characteristics are typical of tumor tissue. (a) 0 h, (b) 24 h, (c) 48 h, (d) 72 h, and (e) 96 h in culture, respectively. H&E staining (40x).

**Figure 3:**
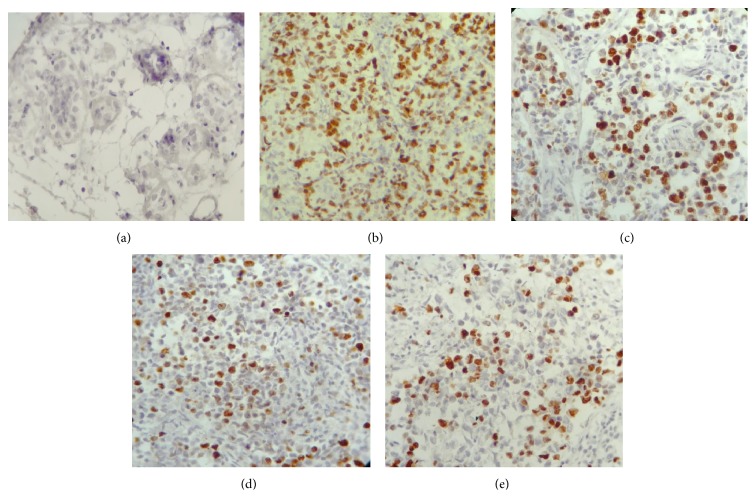
Nuclear expression of the cell proliferation marker Ki 67 in cultures of breast cancer explants. Representative images showing that over 40% of the neoplastic cells express Ki 67 at all-time points. The proliferation index is considered “high” when >20% of the cells are positive for this marker. (a) Normal breast tissue (negative control); (b) breast tumor (positive control); (c), (d), and (e) breast tumor explants cultured for 24, 48, and 72 h, respectively. Immunohistochemical staining (10x).

After 96 h in culture, it was found that the typical histology of the tumor tissue was preserved in breast tumor explants. As shown in Figure 2, in the explants cultured for 24, 48, 72, and 96 h, neoplastic cells retained their characteristic morphology and mitotic activity. It was possible to identify microcalcifications, fibrous connective tissue, desmoplastic stromal reaction, inflammatory cells, and adipose tissue; furthermore, mitotic cells were observed at all-time points. These results (as shown in Figure 3) confirm that breast cancer explants remained viable and actively proliferating for up to 96 h.

#### 3.2.3. Proliferative Activity: Immunohistochemical Expression of Ki 67

Compared to normal breast and tumor tissues (negative and positive controls, resp.), the proliferation index of the cultivated explants was greater than 50% during all the incubation times (24–72 h). This result corresponds to a high proliferation index according to the criteria defined in Materials and Methods and suggests that the tissue remains viable and actively proliferating during* ex vivo* culture conditions (Figure 3).

#### 3.2.4. Effects of Bioactive Compounds and TX on Breast Tumor Explants

**Figure 4:**
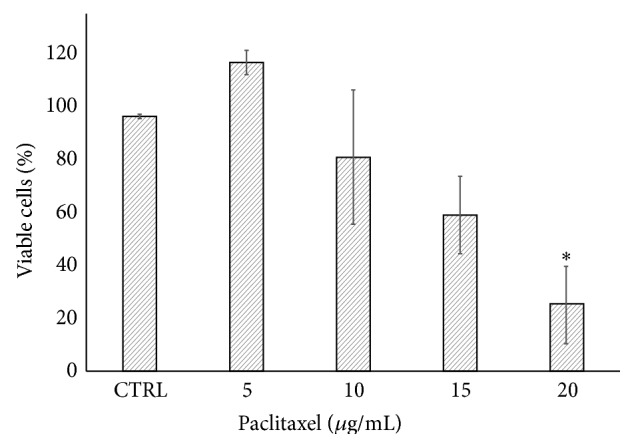
Effect of paclitaxel on the viability of tumor explants. Breast tumor explants were cultured during 48 h in the presence of different concentrations of paclitaxel. The viability was assessed at the indicated times using the Alamar Blue assay. Values reflect means ± SD. Asterisks (∗) indicate significant statistical differences (*P* < 0.05).

In order to ascertain that the breast tumor explants responded to the* ex vivo* treatment with CA, UA, and RA, we decided to first evaluate the metabolic activity of these explants after 48 h of incubation with varying doses of TX (5, 10, 15, and 20 *μ*g/mL). As expected, a dose-response curve of cytotoxicity, directly proportional to the concentration of TX, was observed. Since 20 *μ*g/mL of TX reduced tumor viability to less than 50% (*P* < 0.05), we selected this concentration for assays in which TX was a reference for antineoplastic activity (Figure 4). In the case of the effect of the bioactive compounds on the viability of breast tumor explants, the initial concentrations tested for CA, UA, and RA were 11–33, 20–60, and 20–60 *µ*g/mL, respectively. These concentrations were not cytotoxic since the explants remained viable and also conserved their intact histological structure (data not shown). Because of this, it was necessary to increase the experimental concentrations of CA and UA to 100 *μ*g/mL and of RA to 120 *μ*g/mL.

**Figure 5:**
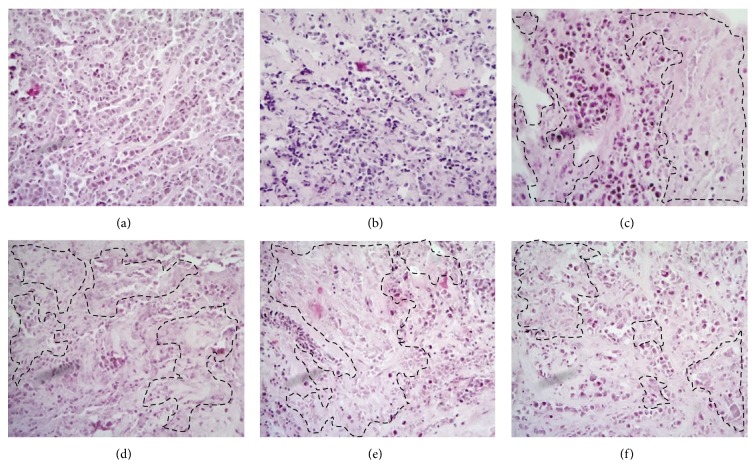
Effect of the bioactive compounds and their combination with paclitaxel on cultures of breast cancer explants. Explants from control at zero h (a) and control without any treatment cultured for 48 h (b) show poorly differentiated invasive neoplasm and no evidence of tumor necrosis areas. In contrast, in explants incubated with TX (c) and their combinations with CA (d), RA (e), and UA (f), an evident pathological response was observed, which is mainly reflected as a notable diminution of the neoplastic cells (40–80%) which can be appreciated as extensive areas of necrosis induced by treatments (dotted lines) H&E staining (10x).

**Figure 6:**
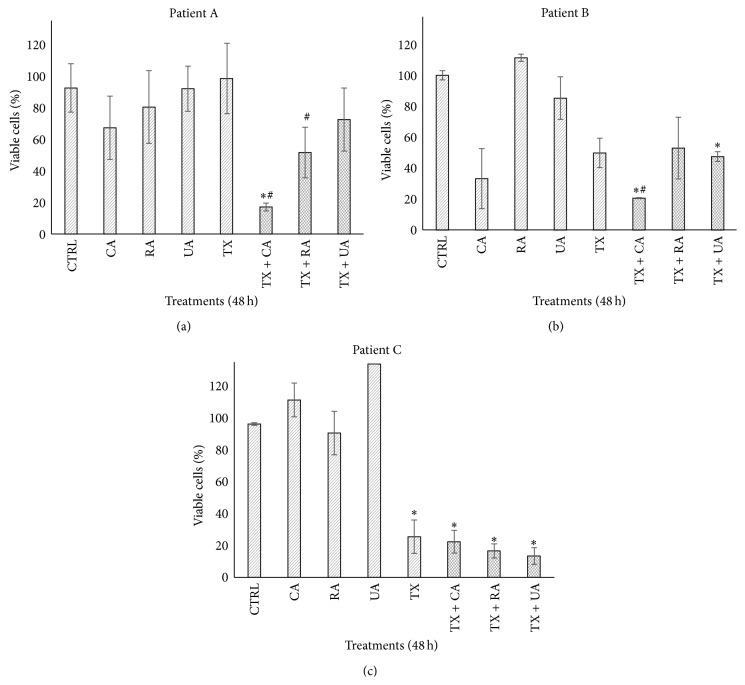
Effect of different treatments on the viability of breast cancer explants. Tumor explants were incubated with the bioactive compounds for 48 h and then cell viability was determined using the Alamar Blue assay. Control explants did not receive any treatment. Concentrations of compounds were 100 *μ*g/mL CA and UA, 120 *μ*g/mL RA, and 20 *μ*g/mL TX. The same concentrations were used in the combinations. Results were compared to the untreated control. Values reflect means ± SD. Asterisks (∗) indicate significant statistical differences (*P* < 0.05) compared to control. Pound key (#) indicates significant statistical differences (*P* < 0.05) compared to paclitaxel.

When the histological structure of uncultured tumor explants was compared with explants cultivated for 48 h without any treatment, poorly differentiated neoplastic with nonspecific pattern neoplastic cells, which retained their viability, were observed. However, when the explants were cultured in the presence of only TX and with combinations of TX plus bioactive compounds, scattered necrotic areas as well as a remarkable reduction (more than 40%) in the population of neoplastic cells were also observed (Figure 5). These results suggest a potential antineoplastic effect of the bioactive compounds, reinforced when they are combined with TX. Additionally, by analyzing the metabolic activity of the explants incubated with these new concentrations, it was observed, as expected, that individual samples from each patient responded differently to the tested compounds. CA was the most effective in patient A, reducing tumor viability to 67.2%, while the combination of TX + CA decreased viability to 17.1%. Likewise, CA was the most active compound against the tumor of patient B, in which viability was reduced by 32.5; also, combination with TX had an important effect on viability, with a reduction of 20.2%. In contrast, none of the compounds had a cytotoxic effect against the patient C sample; however, a marked reduction in viability was observed when individual compounds combined with TX were tested. Interestingly, all of the compounds exerted a synergistic effect, enhancing the tissue toxicity of TX in all three tumor samples (Figure 6).

**Figure 7:**
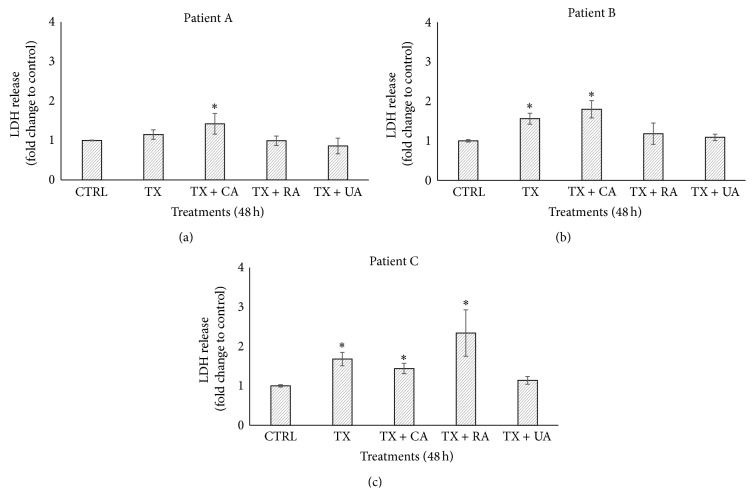
Effect of the combinations of paclitaxel with bioactive compounds on LDH release. Four tumor explants per treatment were incubated with combinations of TX and compounds for 48 h and then LDH activity was quantified by measuring the units of enzyme released into the supernatants from the culture medium. Control explants received no treatment. Concentrations in the combinations were 20 *μ*g/mL + 100 *μ*g/mL (TX + CA and TX + UA) and 20 *μ*g/mL + 120 *μ*g/mL (TX + RA). Results of LDH release are expressed as fold of change relative to control. Values represent means ± SD. Asterisks (∗) indicate significant statistical differences (*P* < 0.05).

The cytotoxicity of TX alone and combinations with bioactive compounds was assessed via the release of the cytosolic enzyme LDH into the supernatants from the culture media in which tumor explants were cultivated. As shown by the data, the combination of TX + CA induced 1.42- and 1.80-fold increase of LDH release in the tumor explants from patients A and B, respectively, compared to untreated control. For patient C, the combination of TX with RA induced a 2.34-fold increase (Figure 7). These values were statistically significant (*P* < 0.05).

## 4. Discussion 

The aim of this study was to demonstrate the use of* ex vivo* organotypic culture of human breast tumor explants as an alternative model system for evaluating natural compounds with antineoplastic potential. The most important characteristic of these explants is that they are obtained from precision-cut breast tissue slices which possess a defined size and thickness.

This is an interesting model which allows the study of different aspects of cancer. It has all the advantages of normal tissues slices and also contributes to the significant decrease in the number of animals used in experimentation [30, 35]. Also, it enables the optimization of the amount of available tissue and favors the realization of a large number of assays that capture many aspects of tumor heterogeneity and complexity [53]. One of the difficulties we faced during the preparation of breast tumor slices was the viscosity or the very soft consistency of some tumors; thus, based on this fact, 2 of the 11 samples were discarded. This disadvantage correlated with those reported by other investigators [54, 55].

To be certain that this system is reliable and adequate to assess the effect of these bioactive compounds, the most critical step was to maintain the viability of the explants during* ex vivo* culture conditions and during subsequent treatment with these compounds. To monitor viability, we used the AB assay because it is a simple and affordable method that allows assessing cell viability by adding AB reagent directly to the culture medium. The active compound of AB is resazurin, which is reduced to resorufin through mitochondrial metabolism in living cells. Moreover the AB assay does not require additional steps as do other viability tests, in which it is necessary to lyse or damage cell membranes in order to release the reduced metabolite [56, 57]. This assay gives reliable measurements of the number of metabolically active cells and is one of the most commonly used methods for assessing cell proliferation. Other advantages include its homogeneous nature, the stability of generated signal, high sensitivity, compatibility with absorbance or fluorescence instruments and different biological models, and also safety for the user and the environment [58, 59]. The results from histopathological and AB analysis demonstrate that both metabolic activity and morphological integrity were conserved for at least 72 h (Figures 1 and 2).

On the other hand, immunohistochemical analysis of Ki 67 showed that cellular proliferation remained stable over the experimental period. Ki 67 is a proliferation marker strongly associated with cells undergoing mitosis in the cellular cycle [60]. Proliferation index in cancer cells from cultivated explants was >40% in all-time points, which is considered “high” according to accepted criteria (Figure 3) [52] and is similar to the reports from other authors, who conducted tests of selective toxicity in breast cancer tissue and were able to maintain viability and proliferation for 24 h [61] and 96 h [44, 62] or up to 7 days [55].

In order to validate the usefulness of the model, we incubated the breast tumor explants with different concentrations of TX, a well-known antineoplastic drug. As was expected, a dose-response curve was observed (Figure 4). With results from three different tumor samples, we used 20 *μ*g/mL as a positive control in the following studies. Afterwards, three independent experiments were carried out to test the effect of CA, UA, and RA, which are naturally occurring products whose anticancer and chemopreventive properties have been reported previously [10, 12, 14, 21, 22, 63, 64]. The concentrations used first were selected on the basis of IC_50_ values reported in cell lines [16, 20, 49]; however, we did not observe a cytotoxic effect on the tumor tissue explants. This can be attributed to the differences between* in vitro* cell cultures and tissue explants, because in tissue explants there is more than one cell lineage interacting with each other and with extracellular matrix components. It is well known that the extracellular matrix and the tumor microenvironment protect neoplastic cells from cytotoxic agents [65]. When we increased the concentrations of the bioactive compounds to 100 *μ*g/mL for CA and UA and 120 *μ*g/mL for RA, we found that CA had the greatest effect, decreasing in tumor viability (Figure 6). The concentration used for CA, as well as the results observed, was similar to those described by Chang et al., who reported that CA induced apoptosis and decreased viability in gastric cancer cells [66].

With regard to the synergistic effect between the bioactive compounds and TX, there are several reports using cell lines which combine antineoplastic agents with extracts from phenolic compounds, such as UA and RA, which enhance treatments effectiveness. These results suggest a great potential for the use of natural compounds, when added to TX or another antineoplastic agent, in order to reduce the dosage, and the side effects associated with chemotherapy, without sacrificing therapeutic results [67–70].

In addition to the aforementioned, performing such studies in organotypic* ex vivo* models, such as the one used in this work, which more closely resemble an* in vivo* scenario, might be more useful for extrapolating results in humans. An important factor to be considered is the administration time of the bioactive compound to the tissue, which can be done before, during, or after incubation with the antineoplastic agent. In our case, we coincubated tumor explants in the presence of compounds plus TX based on the experimental points we previously defined; however, new assays can be designed by pretreating or posttreating tissues with different compounds and antineoplastic agents. When combinations of CA, UA, or RA plus TX were studied, CA acted synergistically with this antineoplastic drug since viability was lower than with TX alone (Figure 6). This result is different from those reported by Lin et al., who found that CA at 100–150 *μ*M induced a slight increase in the proliferation of A549 and H1299 lung cancer cells and that pretreatment of cells with CA protects these cells from growth inhibition when they are incubated with TX [71]. This discrepancy in the results can be attributed to the difference in biological models (cell lines* versus* tumor explants), the pretreatment used, and also the fact that the CA concentrations were different. Furthermore, RA induced more pronounced membrane damage when coadministered with TX in the sample from patient C (Figure 7). Although the synergism between CA and TX was the strongest, a synergistic response in reducing tumor viability for all compounds compared to compounds alone was observed (Figure 6).

The relationship between viability and cytotoxicity data is discrete since, although it is possible to observe that at lower percentage of viability LDH release increases, the values of LDH are relatively low considering that viability decreased at an average of 18% in the synergies between TX + CA in patients A and B and TX + RA in patient C; therefore one would expect LDH levels to be more elevated. One possible explanation for this fact is that inactivation of the enzyme could have occurred in the culture medium, as has been reported by Lash and Zalups, Kendig and Tarloff, Hohnholt et al., and Tulpule et al. [72–75]. Another possibility is that some natural compounds that have antioxidant effects protect cells and prevent the release of LDH. For example, da Silva Morrone et al. found that extracts of* Passiflora manicata* leaves protect from damage induced by reactive oxygen species, and the release of LDH was significantly reduced in precision-cut rat liver slices [76]. On the other hand, Liu et al. found that lipopolysaccharides obtained from* Lycium barbarum* inhibited the elevation of liver enzymes, among them, LDH in slices of liver exposed to carbon tetrachloride [77]. We believe that these last two reports may better explain our findings because these authors also used tissue slices, while, in the reports mentioned above, cell cultures were used. On the other hand, as was described in the introduction, the bioactive compounds we used have antioxidant properties. These findings suggest that inactivation or inhibition of LDH may be more common than previously thought, and investigators should be aware of this at the moment of selecting LDH release as an endpoint for evaluating cytotoxicity.

Taken together, variability in the obtained results is possibly due to the fact that each patient's tumor behaves differently to anticancer drugs, which in turn is due in part to the extensive intratumoral heterogeneity present in each individual tumor [43, 78]. With regard to the last statement, since the 1950s, differential responses to the same drugs in patients with the same histological type of cancer have been reported, including adverse side effects [79]. These differences have been studied in the following years and today it is an accepted fact that individual response to drugs (resistance or sensitivity) depends, among other factors, on the mechanisms of disease (pharmacodynamics), the handling of the drug by patients (pharmacokinetics), the intratumoral heterogeneity, and complex signaling pathways, many of which are still unknown [78, 80, 81]. All these variations are reflected in the intratumoral heterogeneity because of factors that cause genomic instability [29, 78]. Intratumoral heterogeneity and the tumor microenvironment are conserved in the* ex vivo* model we are proposing, and it is possibly one of the reasons why the response to bioactive compounds is different in samples from patients, even when they had breast cancer at the same clinical stage, the same histological type, but different molecular classification.

## 5. Conclusions

In conclusion, our results show that organotypic cultures of breast cancer explants offer an alternative model for the* ex vivo* evaluation of novel compounds with potential anticancer properties, assessing the synergic effect with known anticancer compounds. This model opens perspectives to study biological effects of conventional and innovative treatment strategies in breast cancer research and to analyze different mechanisms of carcinogenesis in other human tumors.

## Figures and Tables

**Table 1:** Bioactive compounds in commonly used herbs and spices.

Caffeic acid	Rosmarinic acid	Ursolic acid
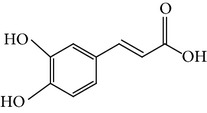	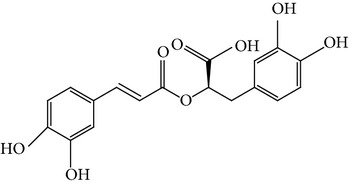	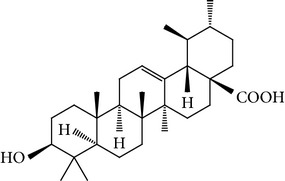
Coffee, parsley	Rosemary	Basil
Cardamom, rosemary	Marjoram	Marjoram
Cumin, sage	Oregano	Sage
Fennel, tarragon	Basil	Thyme
Nutmeg, thyme	Sage	
Oregano	Thyme	

**Table 2:** Clinical and histopathological data of the subjects.

Patient	Age	Clinical stage	Histologic type	Tumor size	Estrogen receptor (ER)	Progesterone receptor (PR)	Her2 status	Molecular classification
A	50 yr	T2N1M0 (grading: IIB)	Ductal infiltrating	3 cm	(−)	(−)	(+)	Her2+
B	59 yr	T3N0M0 (grading: IIB)	Ductal infiltrating	5 cm	(+)	(+)	(+)	Luminal B
C	41 yr	T2N1M0 (grading: IIB)	Ductal infiltrating	4 cm	(+)	(+)	(−)	Luminal A
